# *MYC* break-apart FISH probe set reveals frequent unbalanced patterns of uncertain significance when evaluating aggressive B-cell lymphoma

**DOI:** 10.1038/s41408-021-00578-1

**Published:** 2021-11-24

**Authors:** Marie-France Gagnon, Kathryn E. Pearce, Patricia T. Greipp, Xinjie Xu, Nicole L. Hoppman, Rhett P. Ketterling, Ellen D. McPhail, Rebecca L. King, Linda B. Baughn, Jess F. Peterson

**Affiliations:** 1 Division of Laboratory Genetics and Genomics, Department of Laboratory Medicine and Pathology, Mayo Clinic, Rochester, MN USA; 2 Division of Hematopathology, Department of Laboratory Medicine and Pathology, Mayo Clinic, Rochester, MN USA

**Keywords:** B-cell lymphoma, Cytogenetics

**Dear Editor**,

High-grade B-cell lymphoma (HGBL) with *MYC* and *BCL2* and/or *BCL6* rearrangements is associated with an unfavorable prognosis and poor response to standard chemoimmunotherapy for diffuse large-B-cell lymphoma (DLBCL), prompting the recommendation of more intensive treatment approaches in this setting [1–3]. As this entity is uniquely defined by genetic features and requires a *MYC* rearrangement, it is recommended that B-cell lymphomas with large-cell or high-grade morphology undergo investigation for rearrangements involving this oncogene [4]. Accordingly, fluorescence in situ hybridization (FISH) to identify *MYC* rearrangements in aggressive B-cell lymphomas is routinely performed in clinical laboratories to ensure accurate diagnostic classification and therapy selection.

*MYC* rearrangements in DLBCL and HGBL may involve one of many different partner genes, including the immunoglobulin (IG) heavy chain locus, the kappa or lambda light chain locus, or a non-IG locus. While there is some variability across studies, *MYC* IG partner genes are found in 48–60% of cases and no IG partner is found in 40–52% of cases [5, 6]. Several non-IG rearrangement partners have been described, including *PAX5, BCL6, BCL11A, IKZF1*, and *SOCS1* [7, 8]. Because of the diversity of rearrangement partners and the variability in breakpoints around the *MYC* locus, a break-apart (BAP) FISH probe is optimal to interrogate this gene region as it allows the most reliable detection of both IG and non-IG *MYC* rearrangements. Further refinement of rearrangement partners may be achieved with dual-color dual-fusion (DF) probes spanning *MYC* and *IGH*, IG-lambda (*IGL)* or IG-kappa *(IGK)*. Nonetheless, BAP FISH is the most widely adopted technique to evaluate the *MYC* locus in the clinical laboratory and is often the only FISH assay performed. As a result, the distribution of *MYC* partner genes and associated FISH patterns in DLBCL/HGBL remain underexplored. Of importance, evidence is emerging that the partner gene to which *MYC* is juxtaposed may harbor prognostic significance and high resolution next generation sequencing (NGS) technology demonstrates that the genetic landscape underlying *MYC* rearrangements is more complex than previously recognized [3, 5, 9, 10]. Taken together, these observations underscore the importance of a large-scale appraisal and characterization of *MYC* FISH results in aggressive B-cell lymphomas. In the present study, we describe *MYC* FISH patterns and immunoglobulin rearrangement partners in *MYC-*rearranged (*MYC*-R) aggressive B-cell lymphoma cases investigated at the Mayo Clinic between 2013 and 2017 which had a concurrent BAP and DF evaluation. Herein, we provide the largest-scale portrait of FISH results of *MYC*-R B-cell lymphomas, encompassing more than 930 unselected cases.

This retrospective study was approved by the Mayo Clinic institutional review board. Between August 2013 and December 2017, we routinely performed concurrent BAP *MYC* and DF *MYC*/*IGH*, *MYC*/*IGL*, *MYC*/*IGK* probe sets in paraffin-embedded tissues to detect *MYC* rearrangements for all suspected HGBL cases, for which final diagnoses may have included HGBL with *MYC* and *BCL2* and/or *BCL6* rearrangements, DLBCL, Burkitt lymphoma and HGBL, NOS. Analysis was performed using commercial BAP *MYC* and *MYC*/*IGH* DF probe sets (Abbott Laboratories, Des Plaines, IL, USA) and laboratory-developed DF probes for *MYC*/*IGL* and *MYC*/*IGK* as described by Einerson, et al. [11]. A total of 100 interphase nuclei were analyzed per probe set by two qualified clinical cytogenetic technologists and interpreted by a board-certified (ABMGG) clinical cytogeneticist. The *MYC* BAP probe set includes a red (R) and a green (G) probe which respectively hybridize 5′ and 3′ to the *MYC* gene (chromosomal location 8q24.1), yielding a fusion (F) signal in the setting of an undisturbed gene. In this report, we include *MYC*-R cases as detected by abnormal *MYC* BAP results indicated by balanced (RGF-type pattern) or unbalanced (RF-type or GF-type patterns) rearrangements and concurrent DF results. Unbalanced patterns include loss of a separate green or red signal (such as 1R1F for RF-type patterns or 1G1F for GF-type patterns), suggesting potential loss or gain of material. Cases with *MYC/IGH* fusion identified in the absence of a *MYC* rearrangement by the *MYC* BAP probe were excluded from this study, but have been described by King, et al. [10].

During the four-year study period, a total of 934 independent patient cases were identified to harbor *MYC* rearrangements. Of these, 592 (63.4%) were translocated to an IG partner, whereas no IG partner was identified in 342 (36.6%) cases. BAP FISH revealed typical balanced results (RGF-type pattern) in 823/934 (88.1%) cases. Among the balanced *MYC* rearrangement cases, the DF FISH probes identified an IG partner in 551/823 (67.0%) cases; *IGH* in 415 (50.4%), *IGL* in 102 (12.4%) and *IGK* in 34 (4.1%). The remaining 272/823 (33.0%) balanced *MYC* rearrangements had no IG partner identifiable by DF probe sets. Intriguingly, our data also reveal that a substantial fraction of cases analyzed by the BAP *MYC* assay harbor unbalanced FISH patterns, as identified in 111 (11.9%) cases. Of these, 81 (8.7%) displayed a RF-type pattern and 30 (3.2%) had a GF-type pattern. Among the RF-type pattern, 25 (30.9%) had an IG partner identified with DF probes (16 *IGH*, 5 *IGL* and 4 *IGK*) while 56 (69.1%) had no detectable IG partner. Among the GF-type pattern, *MYC* had an *IGH* partner in 16 (53.3%) cases and no detectable IG partner in 14 (46.7%) cases. No *MYC* rearrangement involving an IG light chain was found in the GF-type pattern, which may be consistent with *IGL/MYC* and *IGK/MYC* rearrangements involving breakpoints located 3’ of *MYC* [11]. The distribution of FISH patterns is detailed in Fig. 1 and in Supplementary Table 1. In comparison with balanced *MYC* rearrangements, unbalanced rearrangements are found at an increased frequency in the setting of no identifiable IG partner (RR 1.91, 95% CI 1.61 to 2.27, *p*-value <0.001), particularly for the RF-type pattern (RR 2.09 95% CI 1.76 to 2.49, *p*-value <0.001) (Supplementary Table 2).

**Fig. 1:**
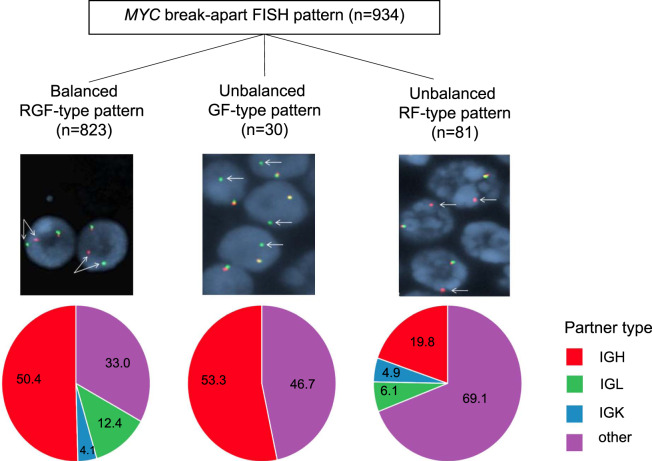
MYC break-apart FISH patterns and frequencies. Representative FISH images of MYC break-apart results, relative frequencies (%) of cases with IG and no IG (other) partners identified by dual-color, dual-fusion FISH probes (from left to right: balanced RGF-type, unbalanced GF-type, unbalanced RF-type patterns).

Few reports have assessed the breadth of *MYC* FISH rearrangement patterns in DLBCL and HGBL. In a cohort including 51 cases of *MYC*-R DLBCL detected by FISH BAP, Copie-Bergman, et al., identified a *MYC/IGH* fusion in 23 cases, a *MYC*/*IGL* fusion in 1 case and no IG partner was observed in 26 cases [5]. In a larger patient cohort by Rosenwald et al., encompassing 264 *MYC*-translocated DLBCL patients, rearrangements involved an IG partner in 107 cases while 88 cases did not display an IG partner [3]. In a previous study by our group including 87 *MYC*-R cases, the rearrangement partner was *IGH* in 39, *IGL* in 7, *IGK* in 6 and no IG partner was observed in 35 cases [6]. However, there is an overall dearth of literature pertaining to atypical *MYC* rearrangements by FISH BAP. Our work calls attention to this phenomenon of uncertain significance and its unexpectedly high frequency. While our observations suggest that some of these unbalanced cases appear to represent true *MYC* rearrangements, as identified by fusion signals with the IG heavy or light chain loci on DF FISH assays, the significance of cases for which no IG partner is identifiable remains unclear (accounting for 7.5% of total cases and 63.0% of unbalanced cases), as these could arise from different genomic alterations such as rearrangements with non-IG partners but also deletions or others. Our study underscores the importance of delineating the genomic mechanisms underlying these atypical FISH findings to allow accurate interpretation of results, especially considering that in multiple myeloma, these have been shown to represent true *MYC* rearrangements [12].

Importantly, the clinical and prognostic significance of atypical *MYC* signals by break-apart FISH assays also remain unresolved. As a large reference clinical genomics laboratory with the inability to obtain comprehensive clinical data for patients treated at other institutions, our study is limited by the absence of outcome data to elucidate potential differential prognostic implications of distinct FISH patterns. Additional work to correlate atypical *MYC* findings with clinical information should be sought. It should also be noted that we solely focused on *MYC* rearrangements as identified by BAP FISH strategy. However, our group and others have previously highlighted false negative findings with *MYC* BAP assays, which may occur at a rate of at least 4% [9, 10, 13–15]. Finally, our study may include cases for which the final diagnosis was not restricted to DLBCL or HGBCL, a limitation which is inherent to investigation algorithms of suspected aggressive B-cell lymphoma.

In summary, our study provides the largest portrayal of *MYC* FISH patterns in aggressive B-cell lymphomas evaluated in paraffin tissue. Importantly, our findings enable appreciation for the existence of frequent unbalanced *MYC* FISH results with the most used FISH strategy, a *MYC* BAP probe, resulting in 11.9% of total *MYC* rearrangement cases with an unbalanced BAP *MYC* result. In addition, the concurrent application of all 3 DF probes (*MYC/IGH*, *MYC/IGL* and *MYC/IGK*), which are not available/applied in most genomics laboratories, still resulted in 7.5% of total *MYC*-R cases with an unbalanced BAP *MYC* FISH result of unclear significance. The genomic alterations leading to these unbalanced FISH patterns should be further explored to guide appropriate interpretation in the clinical laboratory. As a diagnosis of HGBL with *MYC* and *BCL2* and/or *BCL6* rearrangements relies, amongst others, on the identification of a *MYC* rearrangement in a lymphoma with otherwise variable morphology, it is imperative to understand the significance of these atypical FISH findings.

## Supplementary information

Supplementary Table 1

Supplementary Table 2
