# Supplementary material for: MYC break-apart FISH probe set reveals frequent unbalanced patterns of uncertain significance when evaluating aggressive B-cell lymphoma
Source: Blood Cancer J. 2021 Nov 24;11(11):184. doi: 10.1038/s41408-021-00578-1 (PMC8613271; doi:10.1038/s41408-021-00578-1)
Supplement: Supplementary file 1 — Supplementary Table 1 [file 41408_2021_578_MOESM1_ESM.docx]

**Supplementary Table 1.** *MYC* fusion partner as identified by dual-color, dual-fusion probe sets according to signal pattern with *MYC* break-apart FISH

| ***MYC* fusion partner via DF probe set** | **Signal patterns for *MYC* break-apart FISH probe** | | | |
| --- | --- | --- | --- | --- |
|  | **Balanced *MYC* rearrangements (RGF-type pattern) (n=823)** | **Unbalanced *MYC* rearrangement (GF-type pattern) (n=30)** | **Unbalanced *MYC* rearrangement (RF-type pattern) (n=81)** | **Total cases (n=934)** |
| - IGH | 415 (50.4%) | 16 (53.3%) | 16 (19.8%) | 447 (47.9%) |
| - IGL | 102 (12.4%) | 0 (0.0%) | 5 (6.1%) | 107 (11.5%) |
| - IGK | 34 (4.1%) | 0 (0.0%) | 4 (4.9%) | 38 (4.1%) |
| No IG partner identified | 272 (33.0%) | 14 (46.7%) | 56 (69.1%) | 342 (36.6%) |
