# Supplementary material for: MYC break-apart FISH probe set reveals frequent unbalanced patterns of uncertain significance when evaluating aggressive B-cell lymphoma
Source: Blood Cancer J. 2021 Nov 24;11(11):184. doi: 10.1038/s41408-021-00578-1 (PMC8613271; doi:10.1038/s41408-021-00578-1)
Supplement: Supplementary file 2 — Supplementary Table 2 [file 41408_2021_578_MOESM2_ESM.docx]

**Supplementary Table 2**. Relative risk of no detectable IG partner vs IG partner with dual color dual fusion probe sets according to *MYC* BAP balanced or unbalanced pattern

| ***MYC* break-apart FISH probe pattern** | **Relative risk (no IG partner/IG partner)** | **95% confidence interval** | **p-value** |
| --- | --- | --- | --- |
| Balanced | *(ref)* | *(ref)* | *(ref)* |
| Unbalanced | 1.91 | (1.61 , 2.27) | <0.001 |
| Unbalanced (GF-type pattern) | 1.41 | (0.95 , 2.10) | 0.12 |
| Unbalanced (RF-type pattern) | 2.09 | (1.76 , 2.49) | <0.001 |

Chi-square tests were used to assess for between-group differences
